# Genetic Analysis of Central Carbon Metabolism Unveils an Amino Acid Substitution That Alters Maize NAD-Dependent Isocitrate Dehydrogenase Activity

**DOI:** 10.1371/journal.pone.0009991

**Published:** 2010-04-01

**Authors:** Nengyi Zhang, Amit Gur, Yves Gibon, Ronan Sulpice, Sherry Flint-Garcia, Michael D. McMullen, Mark Stitt, Edward S. Buckler

**Affiliations:** 1 Institute for Genomic Diversity, Cornell University, Ithaca, New York, United States of America; 2 Max Planck Institute of Molecular Plant Physiology, Potsdam-Golm, Germany; 3 United States Department of Agriculture-Agricultural Research Service (USDA-ARS) and Division of Plant Sciences, University of Missouri, Columbia, Missouri, United States of America; 4 United States Department of Agriculture-Agricultural Research Service (USDA-ARS) and Department of Plant Breeding and Genetics, Cornell University, Ithaca, New York, United States of America; Michigan State University, United States of America

## Abstract

**Background:**

Central carbon metabolism (CCM) is a fundamental component of life. The participating genes and enzymes are thought to be structurally and functionally conserved across and within species. Association mapping utilizes a rich history of mutation and recombination to achieve high resolution mapping. Therefore, applying association mapping in maize (*Zea mays* ssp. *mays*), the most diverse model crop species, to study the genetics of CCM is a particularly attractive system.

**Methodology/Principal Findings:**

We used a maize diversity panel to test the CCM functional conservation. We found heritable variation in enzyme activity for every enzyme tested. One of these enzymes was the NAD-dependent isocitrate dehydrogenase (IDH, E.C. 1.1.1.41), in which we identified a novel amino-acid substitution in a phylogenetically conserved site. Using candidate gene association mapping, we identified that this non-synonymous polymorphism was associated with IDH activity variation. The proposed mechanism for the IDH activity variation includes additional components regulating protein level. With the comparison of sequences from maize and teosinte (*Zea mays* ssp. *Parviglumis*), the maize wild ancestor, we found that some CCM genes had also been targeted for selection during maize domestication.

**Conclusions/Significance:**

Our results demonstrate the efficacy of association mapping for dissecting natural variation in primary metabolic pathways. The considerable genetic diversity observed in maize CCM genes underlies heritable phenotypic variation in enzyme activities and can be useful to identify putative functional sites.

## Introduction

Glycolysis and the tricarboxylic acid (TCA) cycle, known as CCM, are responsible for the production of accessible energy and the creation of primary building blocks of other metabolisms. Therefore, CCM is critical to plant growth and development. IDH is a TCA cycle enzyme that produces 2-oxoglutarate required by the glutamine synthetase and glutamate synthase cycle for nitrogen assimilation. To study the genetics of CCM, the main focus to date has been on scoring gene expression [1], [2] or metabolite levels [3], [4], [5]. For technical reasons, relatively little attention has been paid to adding protein levels or enzyme activities. However, there is evidence that protein levels and enzyme activities are more heritable because they integrate over time, in a manner analogous to whole plant phenotypes [6]. In this study we adopted a robot-based platform to simultaneously measure multiple enzyme activities [6] in order to study the genetics of CCM enzyme activities in maize.

Linkage analysis and association mapping are two of the most commonly used tools for dissecting complex quantitative traits, known as quantitative trait loci (QTL) mapping. In plants, metabolic QTL has been reported in the studies in tomato [4], *Arabidopsis* [7], [8] and maize [9], [10], [11], [12], [13], [14]. These studies use biparental mapping populations (F_2_, RILs, NILs etc.) that obviously capture only a limited fraction of the variation and do not have sufficient resolution to identify candidate polymorphisms within structural genes. The other drawback lies in the fact that dissecting a quantitative trait down to a gene level using traditional linkage populations could be a long, challenging, and tedious process [15].

Previously, association mapping was used mainly in human genetics, but it is now becoming a common complement to biparental crosses in the genetic mapping of quantitative traits in plants [16], [17], [18]. Among the advantages of this approach are the ability to analyze diverse germplasm that represents multiple alleles for each locus, as well as the utilization of a rich history of mutation and recombination to achieve high resolution mapping.

Maize, a major crop worldwide, is a particularly attractive system for association mapping. Studies have shown that in maize, the advantages of association mapping can be efficiently exploited for starch metabolism in kernels [19], [20], [21], maysin [22], and carotenoids [23]. The enzymes from CCM are conserved at the molecular and functional levels. The availability of extensive genetic diversity is crucial when studying conserved or selected pathways [19], and maize meets this availability requirement because it is the most diverse model crop species. In addition, the unique pattern of linkage disequilibrium (LD) in maize, where LD decays within only a few kb in a population of diverse inbreds, makes it suitable for LD-based, high-resolution association mapping [24].

Searching for the signatures of domestication-related selection among CCM gene sequences will provide useful information to reveal fundamental metabolic properties. In the fish *Fundulus heteroclitus*, clinal variation in lactate dehydrogenase (Ldh, EC1.1.1.27) allozyme allele frequency along the environmental gradient has been found, with the LdhB^b^ allele type having greater catalytic efficiency at low temperatures and the LdhB^a^ allele type having greater catalytic efficiency at high temperatures [25], [26]. Similar studies were also conducted in alcohol dehydrogenase (Adh, EC 1.1.1.1) for *Drosophila* [27], [28]. Clinal variation in allozyme allele frequency along an environmental gradient is often taken as strong, indirect evidence for natural selection. Wright et al. [29] analyzed single-nucleotide polymorphisms (SNPs) in 774 genes in maize and indicated that 2 to 4% of these genes experienced artificial selection. In central starch production in the kernel of maize, four of six genes were selection targets [19]. The proportion of CCM genes under selection still remains to be revealed.

In this project, we used a maize diversity panel and accessions of teosinte to conduct the genetic analysis of CCM. We showed here the presence of substantial genetic variation among various CCM genes. Using association mapping, we identified that this variation in one case is related to heritable enzyme activity variation. We also evaluated selection at CCM loci by examining nucleotide diversity in maize and in its wild ancestor, teosinte. We demonstrated that association mapping combined with other approaches can be an efficient way to dissect natural variation in conserved metabolic pathways.

## Results

### Activities of CCM Enzymes Are Heritable and Co-regulated

We tested if the polymorphisms within CCM genes were associated with variation in the corresponding enzyme activities. A preliminary panel of eight diverse maize inbred lines, grown in five replications, was used to assess the activity variation across nine CCM enzymes. We listed the mean and standard error of activities of the nine CCM enzymes in Table S1. The results showed that all nine enzymes tested from the glycolytic and TCA pathways had substantial differences in activity and significant genetic effects in leaf samples (Table 1). The extent of such heritable activity variation is surprising for core metabolic enzymes, and might indicate the occurrence of selection during adaptation. Positive correlations were found between most enzyme activities (Table 2), suggesting some form of co-regulation acting on these enzymes. Therefore, we also analyzed the residual variation not controlled by co-regulation (i.e. normalized). The normalized heritabilities were still high (Table 1). Based on these analyses and follow-up studies, we focused on IDH, which had the most significant heritable variation.

**Table 1:** Genetic effects and heritabilities of nine CCM enzyme activities.

Enzyme^a^	Genetic effect *P*-value^b^	Raw Heritability^c^	Normalized Heritability^d^
IDH	2.9E-08	0.68	0.40
G6PDH	2.9E-05	0.61	0.57
ALD	4.3E-05	0.58	0.57
PGM	7.0E-04	0.55	0.47
FUM	0.003	0.55	0.33
NADP-IDH	5.7E-05	0.49	0.42
PGK	0.025	0.26	0.16
GLK	0.030	0.25	0.20
GAPDH	0.052	0.22	0.08
**Mean-STD activity**	**5.5E-05**	**0.53**	

Values were calculated from a set of 8 diverse maize inbred lines grown and measured in five replications.

a IDH: NAD-dependent isocitrate dehydrogenase, G6PDH: glucose-6-phosphate dehydrogenase, ALD: fructose-biphosphate aldolase, PGM: phosphoglucomutase, FUM: fumarase, NADP-IDH: NADP-dependent isocitrate dehydrogenase, PGK: phosphoglycerate kinase, GLK: glucokinase, GAPDH: NADP-dependent glyceraldehyde 3-phosphate dehydrogenas.e.

b The taxa effect in a one-way ANOVA.

c Broad-sense heritability, the proportion of the genetic variation from the total phenotypic variation (σ^2^_G_/(σ^2^_G_+σ^2^_E_)).

d Normalized heritability was calculated in the same way as broad-sense heritability but using normalized enzyme activity values that are the difference between each individual standardized activity value of a sample and the mean standardized activity of each sample across the 10 enzymes.

**Table 2:** Correlations between activities of nine CCM enzymes.

	Glycolysis						TCA		
	G6PDH	ALD	PGK	GAPDH	GLK	PGM	NADP-IDH	IDH	FUM
G6PDH									
ALD	−0.09								
PGK	0.03	**0.87**							
GAPDH	0.13	**0.80**	**0.90**						
GLK	−0.07	**0.32**	**0.33**	0.06					
PGM	0.06	**0.77**	**0.80**	**0.73**	**0.39**				
NADP-IDH	0.01	**0.58**	**0.45**	**0.31**	**0.37**	**0.33**			
IDH	**0.43**	**0.64**	**0.50**	**0.55**	0.11	**0.42**	**0.70**		
FUM	**0.58**	**0.41**	**0.39**	**0.30**	**0.44**	**0.46**	**0.41**	**0.57**	

Correlations were calculated from measurements of 40 samples representing 8 lines in 5 replications each. Bolded values: *P*<0.05. Underlined values: *P*<0.001. G6PDH: glucose-6-p dehydrogenase, ALD: fructose-biphosphate aldolase, PGK: phosphoglycerate kinase, GAPDH: NADP-dependent glyceraldehyde 3-phosphate dehydrogenase, GLK: glucokinase, PGM: phophoglucomutase, NADP-IDH: NADP-dependent isocitrate dehydrogenase, IDH: NAD-dependent isocitrate dehydrogenase, FUM: fumarase. TCA: tricarboxylic acid cycle.

### Phe/Tyr^209^ Polymorphism Is Associated with the IDH Activity

**Figure 1:**
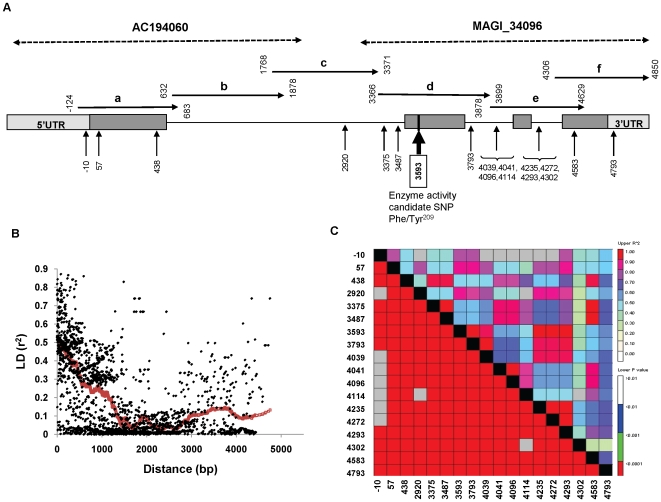
Gene model, LD structure and sequencing coverage of the *idh* gene. (**A**) *idh* gene model. Dark gray boxes represent exons. Light gray boxes are the 5′ and 3′ UTRs. Vertical arrows and numbering below the gene model are the locations of SNPs that showed significant association with IDH activity. Dashed arrows above the gene are the BAC and MAGI contigs that were used as reference sequence for primer design. Horizontal arrows above the gene represent the sequenced amplicons that were used for genotyping. Regions a, c, d, and f were sequenced across 288 maize lines, region e was sequenced across 100 lines, and region b was first sequenced across four lines and then genotyped across the 288 inbred line set. (**B**) LD plot for squared correlations of allele frequencies (*r^2^*) against distance between polymorphic sites within the *idh* gene in 100 lines. **R**ed dots represent average *r^2^*. (**C**) LD plot for the sites of *idh* significantly associated with IDH activity.

Since previous studies showed that activities of Ldh and Adh are affected by both genetic and environmental differences and their G X E interaction in fish *Fundulus heteroclitus* [25], [26] and *Drosophila* [27], [28], respectively, we wanted to see if the same pattern applies to IDH. Therefore, we conducted IDH activity assay at 25°C and at 35°C in 100 diverse inbred lines. However, while the temperature effect on IDH activity was highly significant, there was no genotypic interaction with temperature (Figure S1). Initial association analysis on the two maize IDH paralogs indicated that variation in only one of the paralogs associated with enzyme activity while the second paralog did not (data not shown). Four overlapping regions, covering approximately 3.5 kb of genomic sequence from the IDH gene (the significant paralog), were sequenced across the 288 inbred line set. An additional 0.75 kb region was sequenced across the 100 line subset. This extensive sequence analysis detected 68 SNPs and 17 INDELs with minor allele frequencies (MAF) >0.1 (Figure 1A). The pattern of LD within the *idh* gene decayed relatively quickly (average *r^2^* drops below 0.1 within ∼2 kb), but there were some haplotypes with a longer range LD of 4 kb (Figure 1B). In general, this panel provided gene to subgene resolution for this region of the genome.

**Figure 2:**
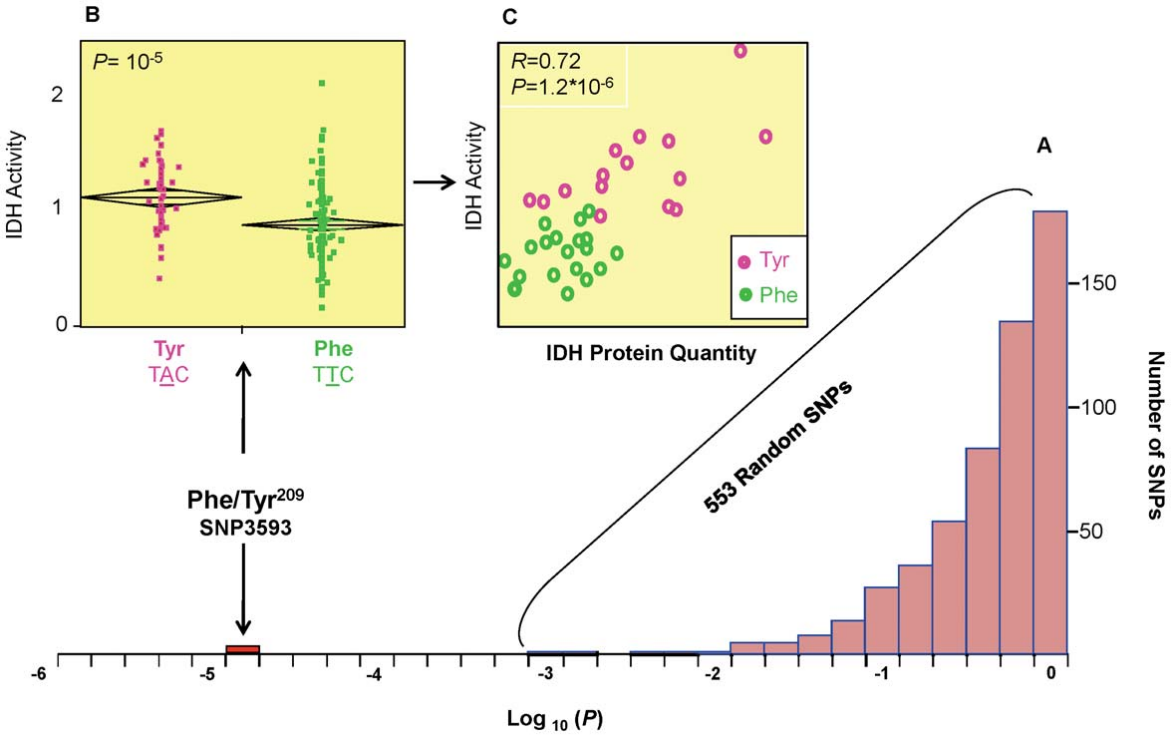
Effect of Phe/Tyr^209^ on IDH enzyme activity and protein quantity. (**A**) Frequency distribution of association tests *P*-values for 553 random SNP (pink bars) and *idh* SNP3593 (red bar). On the X axis are the log_10_ (*P*) values for association tests using mixed-linear model. (**B**) Comparison between the two alleles at SNP3593 for IDH activity. A combined standardized IDH activity data from the two incubation temperatures is presented. (**C**) Correlation between IDH total activity and IDH protein quantity across a subset of 35 lines. Each point represents the mean of 3 biological replications.

Mixed-model association tests that controlled for population structure were performed for all 68 SNPs and 17 INDELs with MAF >0.1 against IDH activity. One of the major concerns in association mapping experiments is the possible detection of non-functional, spurious associations resulting from population stratification [30]. Although there are several methods to control for population structure in association mapping that were also applied here [31], it is still useful to estimate the extent of false positives using a null distribution of association tests *P*-values from a random set of markers. Comparison of the *P*-values obtained from SNPs in a candidate gene to the distribution of *P*-values from random markers can put the candidate SNPs' *P*-values in a more realistic perspective, irrespective to the method that was used to control for population structure. Figure 2A shows the distribution of *P*-values for 553 random SNPs tested against IDH activity together with the *P*-value obtained for IDH SNP3593 under the same rigorous statistic test. The set of 553 random markers that are used here are not expected to capture true associations due to the fast-decaying LD structure in our population. Indeed, the distribution of *P*-values for the 553 random SNP was not different from the expected null distribution.

The association analyses showed that thirteen SNPs and five INDELs from *idh* were significant at *P*<10^-4^ (Figure 1A), which greatly exceeded the significance levels of 553 SNPs in a random panel (Figure 2A). Only one of the significant SNPs (*idh* SNP3593) encoded a non-synonymous polymorphism, resulting in a tyrosine (TAC) (polar amino acid) to phenylalanine (TTC) (non-polar amino acid) substitution (Phe/Tyr^209^). The effect of this SNP on IDH activity was 32% at 25°C and 25% at 35°C (R^2^_SNP_ = 0.11, P = 10^−5^, Figure 2B).

Since all 13 SNPs and 5 INDELs in *idh* showed a similar magnitude of effect, we inspected their LD in an attempt to determine whether they were likely to represent one or more functional sites. The *r^2^* between almost every pair of significant SNPs was higher than 0.4 (Figure 1C). Only a limited number of recombinations were found, creating three major haplotypes and several rare ones. While this LD and haplotype structure restricted the mapping resolution, it was consistent with the SNPs being in LD with one causative polymorphism. Therefore, we hypothesized that Phe/Tyr^209^ is most likely the functional SNP. Further statistical support was provided by the finding that none of the other significant SNPs improved the *R* ^2^ value when tested in a stepwise model alongside Phe/Tyr^209^.

**Figure 3:**
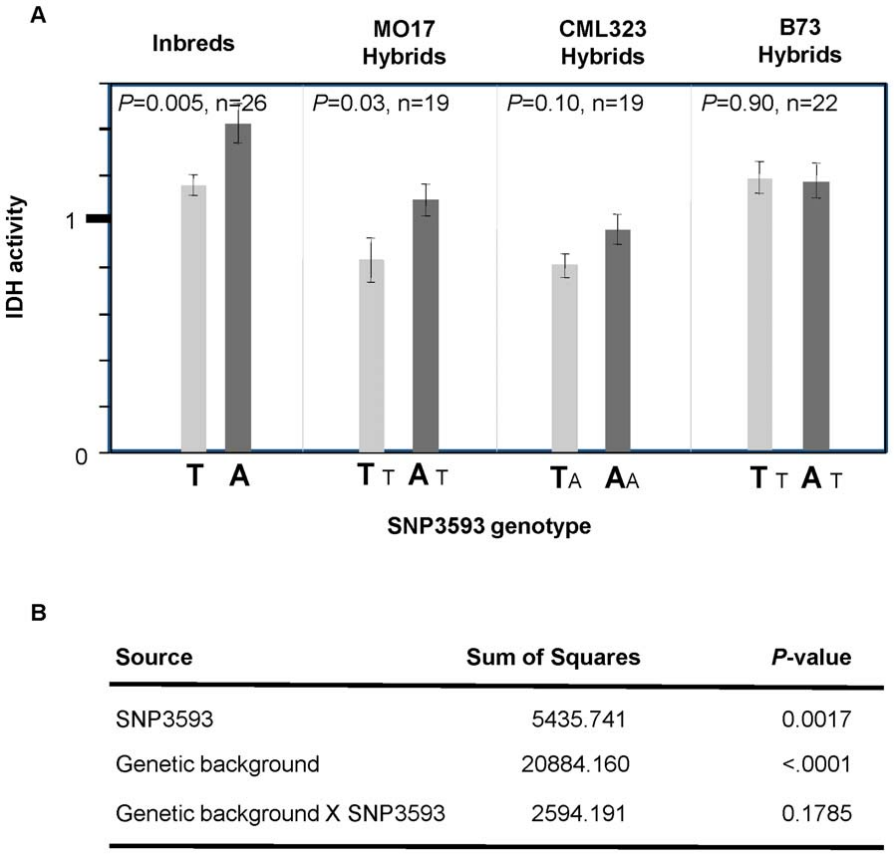
*idh* SNP3593 effect across different genetic backgrounds. (**A**) One-way analysis for *idh* SNP3593 effect at four different genetic backgrounds (inbreds, B73 hybrids, CML323 hybrids and Mo17 hybrids). Bolded letters below each bar represent the allele of the inbred parents. Smaller fonts T and A nucleotides indicate the donated tester allele. IDH activity is presented as standardized values. (**B**) Two-way ANOVA for SNP3593 effect, genetic background effect and the SNP by genetic background interaction.

To further investigate this QTL we tested the effect of the Phe/Tyr^209^ SNP on IDH activity at 25°C in a separate experiment across 26 diverse inbreds and their F_1_ hybrids, with 3 different testers from different germplasm groups. Combined two-way ANOVA revealed significant Phe/Tyr^209^ and genetic background effects (*p* = 0.0017 and *p*<0.0001, respectively), but a non-significant Phe/Tyr^209^ by genetic background interaction (Figure 3B). A significant Phe/Tyr^209^ effect was found across the inbreds (*p* = 0.005) and across hybrids with the Mo17 tester (*p* = 0.03, Figure 3A). A similar trend (*p* = 0.1) was found across the CML323 hybrids. However, the effect in B73 hybrids was not significant, suggesting a weak interaction. These results confirm the basic results and suggest that while background has an effect on this gene, there is no statistical interaction with that background.

Normalization of the IDH activity data by genetic background allowed us to test the mode of action of Phe/Tyr^209^ across this diverse sample. The mode of action was not significantly different from additive; the heterozygote value fell between both homozygous classes, and the dominance effect was not significant (Table 3 and 4).

**Table 3:** IDH activity for three genotypic categories at SNP3593.

SNP3593 Genotype	N	IDH activity
AA_a_	16	0.6182
AT_b_	29	0.0302
TT_b_	41	−0.2626

IDH activity was calculated based on standardized IDH activity data from 26 inbreds and 61 hybrids from three different genetic backgrounds. Genotypic categories with different letters (a or b) are significantly different at *P*<0.01.

**Table 4:** Genetic effects of SNP3593 for IDH activity.

Factor	Effect	*P*-value
A	0.4404	0.002
D	−0.1476	N.S.
d/a	−0.3350	

Mode of action was calculated based on standardized IDH activity data from 26 inbreds and 61 hybrids from three different genetic backgrounds. a: additive effect; calculated as half of the difference between the means of lines homozygous for each allele. d: dominance deviation; calculated as the difference between the mean of lines heterozygous for SNP352 and the midpoint value of the two homozygous classes.

### IDH Protein Quantity Also Contributes to the IDH Activity Variation

**Figure 4:**
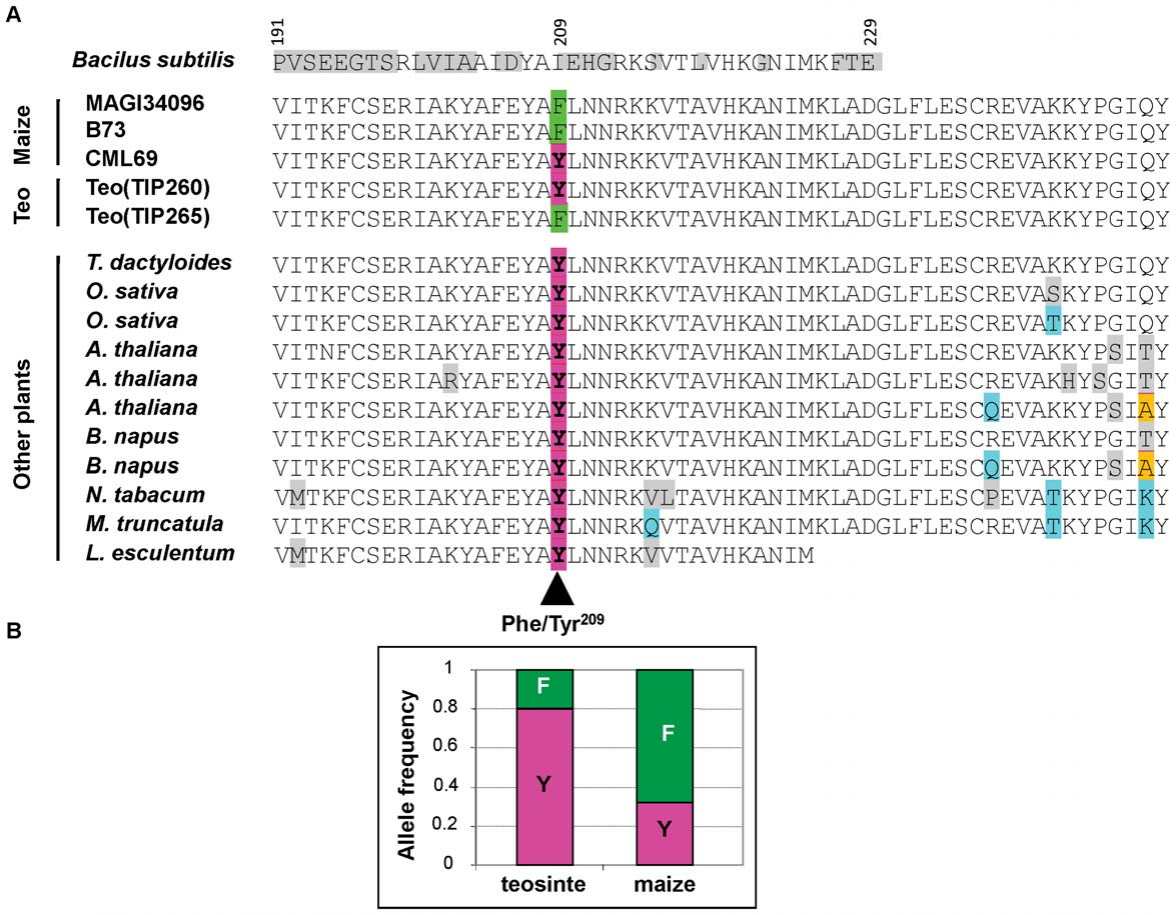
Conservation of Phe/Tyr^209^ across related plant species and allele frequencies in maize and teosinte. (**A**) Protein sequence alignment of the part of IDH containing Phe/Tyr^209^ SNP. (**B**) Comparison of allele frequencies at Phe/Tyr^209^ between maize and teosinte. The teosinte sample contained 16 different accessions and the maize sample contained 267 inbred lines. *P*(*χ* ^2^)  = 0.004 for the comparison.

The Tyr^209^ residue was completely conserved in *idh* orthologs in other sequenced plant species (Figure 4A), as were most of the surrounding residues in this region. Alignment of the maize IDH protein with those of other organisms for which the IDH crystal structures have been defined allowed us to predict that Phe/Tyr^209^ is located on the protein surface, but not in the active site [32].

To address the biochemical mode of action of this quantitative trait nucleotide (QTN), we quantified IDH protein content for a subset of 35 selected lines carrying different alleles at Phe/Tyr^209^, using quantitative scanning of immunoblotted gels. IDH protein quantity was significantly different between the two Phe/Tyr^209^ alleles (P = 8.3×10^−7^). There was a positive correlation between protein quantity and enzyme activity (*R* = 0.72; Figure 2C). This result suggests that at least some of the variation in total IDH activity is explained by differences in IDH protein quantity.

We re-tested the Phe/Tyr^209^ effect on IDH activity after normalizing the activity for IDH protein quantity. The SNP effect on the normalized specific activity was still significant (*P* = 3.6×10^−7^), suggesting that at least two independent factors contribute to the variation in total activity. A stepwise regression where both protein content and Phe/Tyr^209^ were used as factors in the model against IDH total activity confirmed that both independently contribute to the activity variation, with the Phe/Tyr^209^ effect being stronger (Table 5).

**Table 5:** Two-way ANOVA of IDH enzyme activity.

Source	Sum of Squares	F Ratio	*P*-value
SNP3593	2.14	55.02	2.4E-08
Protein quantity	0.29	7.54	0.0100

Based on these results we suggest a two-factor model to explain the variation in IDH total activity; the first component is most likely an element regulating IDH protein level and the second component is Phe/Tyr^209^, which most likely affects enzymatic properties including the specific activity.

### Low-activity Allele (Phe^209^) of IDH Is More Frequent in Domesticated Maize

The Phe/Tyr^209^ residue segregates in maize and also in its wild ancestor, teosinte (Figure 4B). The frequency of the tyrosine allele is still high among teosinte accessions (Freq of Tyr  = 80%, N = 16) but is substantially reduced among maize inbreds (Freq of Tyr = 30%, N = 267; P(χ^2^) = 0.004) (Figure 4B). No significant difference in allele frequency was found between tropical and non-tropical maize germplasm (data not shown). This significant shift in allele frequency between teosinte and maize could be a result of selection. Our data therefore indicate that the low-activity allele (Phe) was favored among domesticated maize. A negative correlation between IDH activity and plant biomass was also identified in a separate experiment across 60 diverse maize hybrids in five replications (*R* = −0.46, *P* = 0.0002) suggesting that reduced IDH activity in leaves can have a positive effect on growth rate and, thus, can be a target for indirect selection.

### Some CCM Genes Had Been Targeted for Selection during Maize Domestication

In addition to directly testing for associations between polymorphisms within CCM genes with enzyme activities, another way to reveal fundamental metabolic properties is to search for the signatures of domestication-related selection among CCM gene sequences, as previously shown across a large set of random genes in maize [29]. Here, we sequenced 17 CCM loci across 28 diverse maize inbred lines and 16 teosinte accessions and examined nucleotide diversity in maize relative to that in teosinte (Table 6). The pi-maize vs. pi-teosinte ratio for CCM genes was compared with that of random genes [29] (Table 6).

**Table 6:** CCM gene diversity in maize and teosinte.

Locus	pi-Maize	pi-Teosinte	pi-Maize/pi-Teosinte	Percent tail in random genes
*Ald*	0.0010	0.0066	0.1451	14^a^
*eno2*	0.0001	0.0009	0.1474	14
*Scoal*	0.0011	0.0069	0.1560	14
*Fum*	0.0041	0.0079	0.5186	36
*Idh*	0.0032	0.0058	0.5466	38
*g6pdh*	0.0072	0.0106	0.6766	51
*eno*	0.0095	0.0118	0.8071	64
*hex*	0.0087	0.0107	0.8161	64
*pgam*	0.0049	0.0058	0.8495	68
*pgm*	0.0108	0.0126	0.8608	69
*sdh*	0.0052	0.0054	0.9527	76
*mdh*	0.0087	0.0083	1.0560	82
*Ald2*	0.0095	0.0083	1.1392	87
*pgk*	0.0092	0.0078	1.1829	89
*aco*	0.0051	0.0039	1.2811	91
*pgm2*	0.0092	0.0063	1.4655	93
*ogdh*	0.0040	0.0026	1.5768	94
average	0.0059	0.0072	0.8234	65

a The corresponding percent tail of pi-Maize vs. pi-Teosinte ratio in 774 random genes [29] for the CCM gene.

*aco*: *aconitase*, *ald or ald2*: *fructose-biphosphate aldolase*, *eno* or *eno2*: *enolase*, *fum*: *fumarase*, *g6pdh*: *glucose-6-phosphate dehydrogenase*, *hex*: *hexokinase*, *idh*: *NAD-dependent isocitrate dehydrogenase*, *mdh*: *NADP-dependent malate dehydrogenase*, *ogdh*: *oxoglutarate dehydrogenase*, *pgam*: *phosphoglycerate mutase*, *pgk*: *phosphoglycerate kinase*, *pgm* or *pgm2*: *phophoglucomutase*, *scoal*: *succinyl-CoA ligase*, *sdh*: *succinate dehydrogenase*.

The results showed that CCM genes were within normal range of bottleneck associated domestication. One of our 17 CCM genes, *hexokinase* (*hex*), was also included in the 774 random genes and is one of the 30 selected genes [29]. However, we did not find selection signature on it. The discrepancy could be explained by the different regions sequenced and different maize lines and teosinte accessions used. Another CCM gene, *aconitate hydratase* (*aconitase, aco*), which is a different paralog from the one we studied in the 17 CCM genes, is also one of the 30 selected genes [29] though we did not find artificial selection signature on the paralog we studied. Therefore, like other genes being selected, some CCM genes had also been targeted for selection during maize domestication.

In addition, we found the presence of teosinte-specific non-synonymous (results not shown) SNPs that can be tested for their association with enzyme activity. Such an approach can be a direct way to identify domestication-related metabolic QTNs.

## Discussion

In this study, we used an association-mapping strategy that utilizes diverse, multi-allelic germplasm in a single mapping experiment. For conserved metabolic pathways such as glycolysis and the TCA cycle, such an approach is necessary in order to capture enough functional diversity within candidate genes. We were able to zoom-in to the gene level and identify a putative functional SNP that is associated with variation in a metabolic quantitative trait.

We found that CCM enzyme activities were positively correlated, therefore suggesting some form of co-regulation acting on these enzymes. Moreover, CCM enzyme activities showed great genetic effects and relatively high heritabilites. Similar results were also shown in other studies [7], [8], [33]. In addition, enzyme activities may have simpler mode of genetic control than other complex quantitative traits [7]. Therefore, enzyme activities were mappable traits, ideal for studying the mechanism of underlying QTL [7].

In this study, we identified a novel amino-acid substitution in a phylogenetically conserved site (Phe/Tyr^209^) which was strongly associated with changes in IDH activity. Given its conservation across plants, this amino acid is extremely likely to play an important role in the enzyme. Similar large amino acid changes are responsible for phenotypic variation in sweet corn [19], [21]. Moreover, we found that IDH protein quantity also contributed to the IDH activity. Since SNP3593 resulting in Phe/Tyr^209^ is in LD with the potential transcription related site SNP10 (Figure 1C), it is possible that the protein level difference is due to polymorphisms in the promoter that are in LD with SNP3593. To test this hypothesis, we need to look at the transcription levels and sequence *cis*-regulatory regions (such as the promoter region) to see if the transcript abundance is correlated with protein levels and if the functional sequence variations in *cis*-regulatory regions are in LD with SNP3593. However, predicting protein quantity from transcriptional levels is not that straightforward sometimes because of post-transcriptional, translational or post-translational modification. For example, Gibon et al. [6] found that there was no relation between the amplitudes of the diurnal changes of transcript and enzyme activity for many Arabidopsis enzymes. Nevertheless, neither IDH protein quantity nor Phe/Tyr^209^ alone could account for all the IDH activity. Therefore, we proposed a two-factor model to explain the variation in IDH total activity: an element regulating IDH protein level and a second element, Phe/Tyr^209^, affecting enzymatic properties.

The SNPs significantly associated with IDH activity are in LD and span across the whole gene. Similar allele series are present in the maize *Lycopene Epsilon Cyclase* (*lcyE*) for carotenoid metabolism [23]. Fine mapping within the 4.8 kb of *idh* is still very challenging, but teosinte introgression (Flint-Garcia in prep), teosinte association [34], [35], or landraces will provide more recombination to evaluate these biochemical hypotheses. Recently, the Maize Diversity Project (www.panzea.org) developed the maize nested association mapping (NAM) panel for dissecting complex quantitative traits with high resolution and statistical power [36]. The NAM design integrates linkage and association approaches, by using a population that is comprised of 5000 recombinant inbred lines (RIL) derived from crosses between B73 and 25 highly diverse maize lines (200 RILs for each cross) [36]. In the NAM panel, there are nine of 25 populations segregating for the *idh* SNP3593 and its associated haplotype (data not shown). Therefore, it's possible to use the NAM panel to dissect the IDH activity.

While Wright et al. (2005) [29] found that 2 to 4% of 774 maize genes experienced artificial selection and Whitt et al. (2002) [19] showed that four of six genes in the central starch production pathway in maize kernels were targets of selection, our study showed that some CCM genes had also been targeted for selection during maize domestication process. We detected significant Phe/Tyr^209^ allele frequency shift in *idh* between teosinte and maize. The low-activity *idh* allele (Phe) was favored among domesticated maize and a negative correlation between IDH activity and plant biomass was also identified. Previous studies in tomato showed that increased plant growth as a result of reduced activities of two other TCA enzymes: mitochondrial malate-dehydrogenase [37] and aconitase [38]. The negative correlation between IDH activity and plant growth may be due to increase in photosynthesis, a compensation for the reduction in energy production by respiration [37] or “over” compensation by other isoforms of IDH. Though we found no significant G x E effects for IDH activity in the present study (Figure S1), the reason that CCM genes were targets of selection might still be because of changing temperatures and/or a centralization of the source-sink relationships during domestication. Temperatures in Mexico differ greatly from environments throughout North America and South America. Unlike teosinte, which has long lateral branches terminated by male tassels, maize concentrates all its resources in one inflorescence, and maize has little competition. This suggests that if all CCM enzyme activities parallel IDH, maize CCM activity may be reduced relative to teosinte.

The maize panel used in this study represents a considerable portion of the genetic diversity found in the domesticated maize breeding germplasm [24]. Our results have demonstrated that association mapping is an efficient way to study the genetics of CCM and suggested that there is still enough genetic diversity and phenotypic variation among those enzyme activities and gene sequences in maize. With emerging and anticipated improvements in proteomics and enzymatic measurements for throughput and accuracy, along with robust genotyping platforms, scientists will soon be able to use natural variation to better understand the genetics of metabolism and the importance of metabolism to plant growth and development.

## Materials and Methods

### Plant Materials and Green-house Experiment

For the enzymatic measurements, a preliminary panel of eight diverse maize inbred lines, grown in five replications, was used to assess the phenotypic variation across nine CCM enzymes (Table 1). A subset of 100 inbred lines was selected from the core 300-line association-panel [24] for association mapping. Line selection was based on genotypic data from 553 random SNPs in order to capture maximal genetic diversity and was conducted using the PowerMarker software [39]. The hybrid experiment included 26 diverse inbreds that were selected from the core set to capture maximal genetic diversity. Each of these inbreds was crossed with 3 different testers (B73, Mo17 and CML323) to create F1 hybrids. For enzymatic measurements, plants were grown in five replications in cell-packs in the green-house in a completely randomized design. Three seeds from each line were sown in each cell and thinned five days after germination to one plant per cell to ensure uniform germination across the experiment. Tissue was collected from the youngest expanded leaf 35 days after germination and immediately frozen in liquid nitrogen. Tissue was stored at -80°C until analysis.

### Enzymatic Assays

The activities of the nine CCM enzymes in Table 1 (their pathway locations demonstrated in Figure S2) were measured across the preliminary panel of eight diverse maize inbred lines. Glucose-6-phosphate dehydrogenase (G6PDH, EC 1.1.1.49), fructose-1,6-bisphosphate aldolase (ALD, EC 4.1.2.13), fumarase (FUM, EC 4.2.1.2), NADP-dependent isocitrate dehydrogenase (NADP-IDH, EC 1.1.1.42), glucokinase (GLK, EC 2.7.1.1) and NADP-dependent glyceraldehyde-3-phosphate dehydrogenase (GAPDH, EC 1.2.1.9) were determined as previously described by Gibon et al. [6], phosphoglucomutase (PGM, EC 5.4.2.2) as in Manjunath et al. [40], and phosphoglycerate kinase (PGK, EC 2.7.2.3) as in Huege et al. [41]. NAD-dependent isocitrate dehydrogenase (IDH, EC 1.1.1.41) activity was assayed further across the 100 inbred lines and three groups of hybrids in conditions adapted from McIntosh et al. [42]. Extracts, as well as NADH standards ranging from 0 to 1 nmol, freshly prepared in an extraction buffer [6] containing 20% (v/v) glycerol, 1% (v/v) Triton-X100 and 0.5 mM dithiothreitol were incubated in a medium containing: 50 mM MOPS/KOH pH 7.5; 5 mM MgSO_4;_ and 2 mM NAD^+^. The reaction was started by the addition of isocitrate to a final concentration of 0 (blank) or 4 mM (‘maximal’ activity). The reaction was stopped with 20 µl of 0.5 M NaOH. NADH was then determined as in Gibon et al. [6]. For the 100 inbred line panel each sample was divided and measured for enzymatic activity under two incubation temperatures (25°C and 35°C) to address the presence of G×E interaction. Measurements were performed in 96-well plates where each sample was measured for V_max_ and V_blank_ in the same plate. Each plate contained 40 experimental samples, as well as 4 common reference samples and 4 blank samples for normalization of the data. Raw enzyme activity data was standardized in two ways: 1) by plate mean; and 2) by common reference samples mean. Both methods yielded similar quality of data standardization.

### Protein Quantification

Rabbit antibodies raised against IDH were generously provided by Dr. Michael Hodges (IBP Orsay, France). Protein extraction of IDH for blotting and procedures for gels were performed as in Hendriks et al. [43], except dithiothreitol was added to the sample buffer. Quantification was performed with a Li-Cor Odyssey.

### Sequence Analyses

Sequencing and SNP discovery were conducted as previously described by Whitt and Buckler [44]. One or more amplicons (400–1600 bp each) was sequenced in each of the 17 selected CCM loci across 28 diverse maize inbred lines and 16 teosinte accessions (scheme of the corresponding enzymes of the selected genes and their pathway locations demonstrated in Figure S2 and EMBL/GenBank accession numbers of the amplicons listed in File S1).

Four overlap regions, a, c, d, and f, covering approximately 3.5 kb of genomic sequence from the IDH gene were sequenced across the 288 inbred line set selected from the core 300-line association-panel [24], and an additional 0.75 kb region, e, was sequenced across the 100 lines subset (Figure 1A). The 1.2 kb region, b, was first sequenced across four lines and then genotyped across the 288 inbred line set.

Pairwise LD for *idh* was calculated as *r^2^* between all SNPs pairs (Figure 1B), and nucleotide diversity, π, the average number of nucleotide differences per site between two sequences, for CCM genes was estimated using the TASSEL software [45].

### Statistical Analyses

In order to evaluate the proportion of the co-regulation factor in the observed enzymatic activity variation, we calculated the heritability of the average standardized activity across the nine enzymes. The genetic variation for this overall mean-activity was highly significant and explained 53% of the total phenotypic variation (Table 1), indicating that co-regulation is a major component in the observed enzyme activity variation. In order to determine the remaining enzyme-specific variation (which is the relevant variation for the candidate-gene association mapping), we calculated the normalized, enzyme-specific activity for each enzyme as the difference between the standardized enzyme activity and the overall mean-activity.

All association tests were carried out using TASSEL software [45]. We used the mixed-linear model (MLM) function that accounts for different levels of relatedness and controls the type I error rates as described by Yu et al. [31]. Population structure was also included in the association analyses to eliminate non-functional, spurious associations resulting from population stratification [30]. Trait heritabilities, ANOVAs and Pearson correlations were calculated using JMP5.1 software (SAS institute).

## Supporting Information

Table S1 Activities of nine CCM enzymes for eight different maize inbred lines in five replications.
(0.04 MB DOC)

Figure S1 IDH activity at two incubation temperatures across 100 maize inbred-lines. (A) Correlation between IDH activity at 25°C and 35°C. Each point represents the mean of five replications from each inbred line. Parallel to the X and Y axes are the frequency distribution of the activity measurements. (B) Two way ANOVA for IDH activity against line and incubation temperature.
(0.12 MB TIF)

Figure S2 Selected CCM enzymes and their pathway location. PGM: phophoglucomutase, HEX: hexokinase, GLK: glucokinase, G6PDH: glucose-6-phosphate dehydrogenase, ALD: fructose-biphosphate aldolase, GAPDH: NADP-dependent glyceraldehyde-3-phosphate dehydrogenase PGK: phosphoglycerate kinase, PGAM: phosphoglycerate mutase, ENO: enolase, ACO: aconitase, IDH: NAD-dependent isocitrate dehydrogenase, OGDH: oxoglutarate dehydrogenase, SCoAL: succinyl-CoA ligase, SDH: succinate dehydrogenase, FUM: fumarase, MDH: NADP-dependent malate dehydrogenase. a: Enyzme activities were measured. b: Corresponding loci were sequenced.
(0.13 MB TIF)

File S1 EMBL/GenBank accession numbers.
(0.03 MB DOC)
