# Supplementary material for: Genetic Analysis of Central Carbon Metabolism Unveils an Amino Acid Substitution That Alters Maize NAD-Dependent Isocitrate Dehydrogenase Activity
Source: PLoS One. 2010 Apr 1;5(4):e9991. doi: 10.1371/journal.pone.0009991 (PMC2848677; doi:10.1371/journal.pone.0009991)
Supplement: Table S1 — Activities of nine CCM enzymes for eight different maize inbred lines in five replications. (0.04 MB DOC) [file pone.0009991.s001.doc]

**Table S1.** Activities of nine CCM enzymes for eight different maize inbred lines in five replications.

| Maize line | IDH | G6PDH | ALD | PGM | FUM | NADP-IDH | PGK | GLK | GAPDH |
| --- | --- | --- | --- | --- | --- | --- | --- | --- | --- |
| B73 | 118^a^±10^b^ | 764±49 | 8625± 751 | 2647±168 | 804± 60 | 496±15 | 79800± 4741 | 72±15 | 10377±1030 |
| CML247 | 60±12 | 762±40 | 7411± 610 | 3269±243 | 736± 47 | 369±12 | 88451± 7646 | 86±15 | 11771±1247 |
| Ky21 | 79± 9 | 934±49 | 5915± 863 | 2476±203 | 831± 84 | 334±16 | 76625± 8648 | 69±17 | 10021±1615 |
| Mo17 | 50± 8 | 623±56 | 5662± 596 | 1931±214 | 536± 35 | 331±20 | 63985± 7403 | 33±13 | 8737±1057 |
| Mo18w | 103± 9 | 722±58 | 9187± 811 | 3095±238 | 784± 61 | 385±24 | 95093± 3813 | 116±35 | 13103± 911 |
| NC350 | 70±15 | 670±29 | 5907± 761 | 2804±494 | 795± 59 | 368±33 | 69243± 9340 | 84±26 | 8889±1489 |
| Oh43 | 72±14 | 432±56 | 10958±1141 | 3336±402 | 824±101 | 422±74 | 96287±10165 | 149±36 | 10472± 829 |
| P39 | 9± 3 | 593±33 | 5057± 476 | 2239±129 | 594± 47 | 307±27 | 71908± 6456 | 104±11 | 7700± 681 |
|  |  |  |  |  |  |  |  |  |  |
| Overall | 75± 6 | 703±26 | 7218± 374 | 2668±109 | 730± 26 | 379±13 | 79218± 2853 | 84± 8 | 10125± 456 |

^a^ Mean enzyme activity. The enzyme activity unit is expressed as nanomoles of substrate converted per gram of fresh maize leaf tissue per minute.

^b^ Standard error.

IDH: NAD-dependent isocitrate dehydrogenase, G6PDH: glucose-6-phosphate dehydrogenase, ALD: fructose-biphosphate aldolase, PGM: phosphoglucomutase, FUM: fumarase, NADP-IDH: NADP-dependent isocitrate dehydrogenase, PGK: phosphoglycerate kinase, GLK: glucokinase, GAPDH: NADP-dependent glyceraldehyde 3-phosphate dehydrogenase
