# Supplementary figures and images for: Genetic Analysis of Central Carbon Metabolism Unveils an Amino Acid Substitution That Alters Maize NAD-Dependent Isocitrate Dehydrogenase Activity
Source: PLoS One. 2010 Apr 1;5(4):e9991. doi: 10.1371/journal.pone.0009991 (PMC2848677; doi:10.1371/journal.pone.0009991)

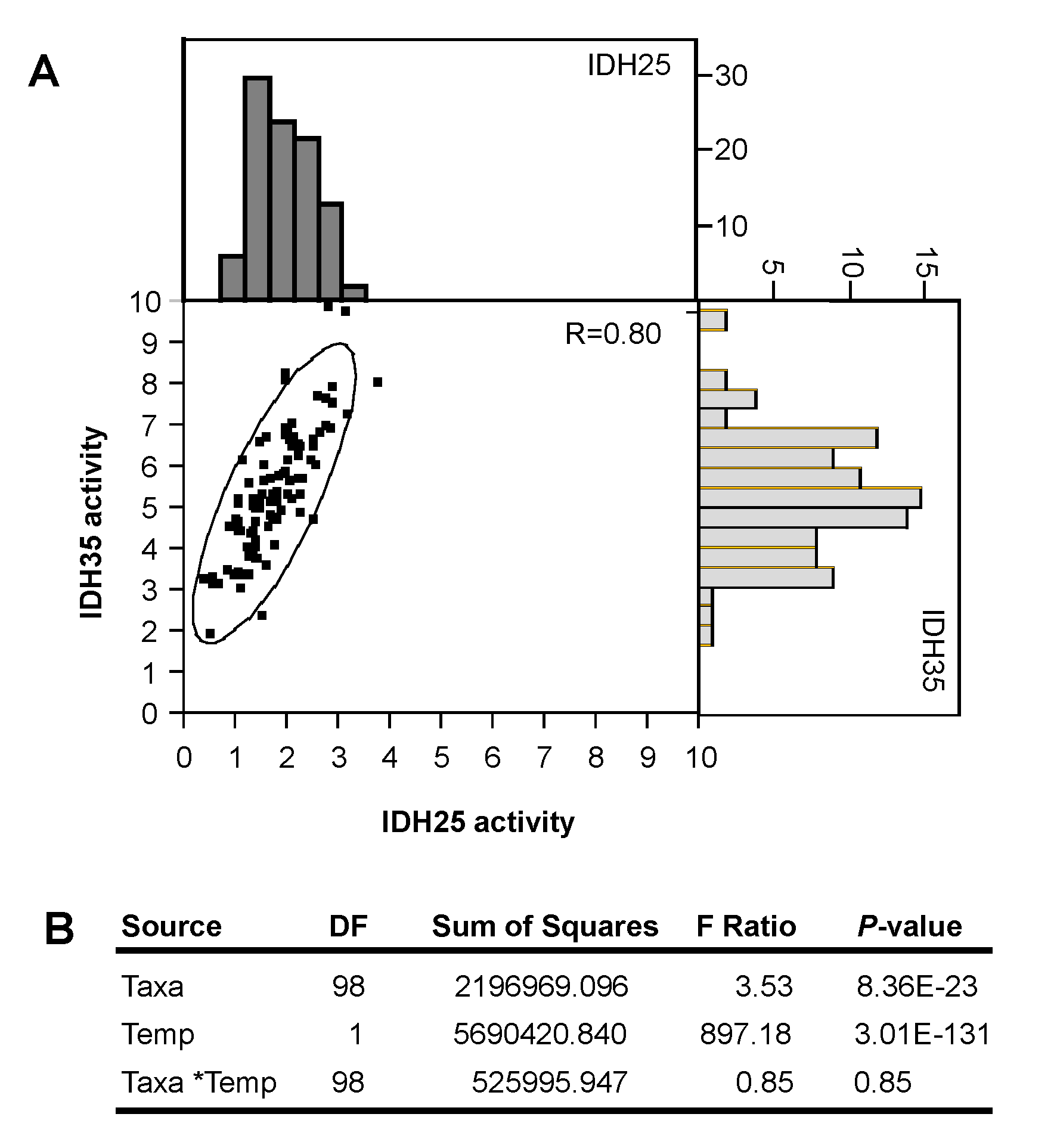

Supplement: Figure S1 — IDH activity at two incubation temperatures across 100 maize inbred-lines. (A) Correlation between IDH activity at 25°C and 35°C. Each point represents the mean of five replications from each inbred line. Parallel to the X and Y axes are the frequency distribution of the activity measurements. (B) Two way ANOVA for IDH activity against line and incubation temperature. (0.12 MB TIF) [file pone.0009991.s002.tif]

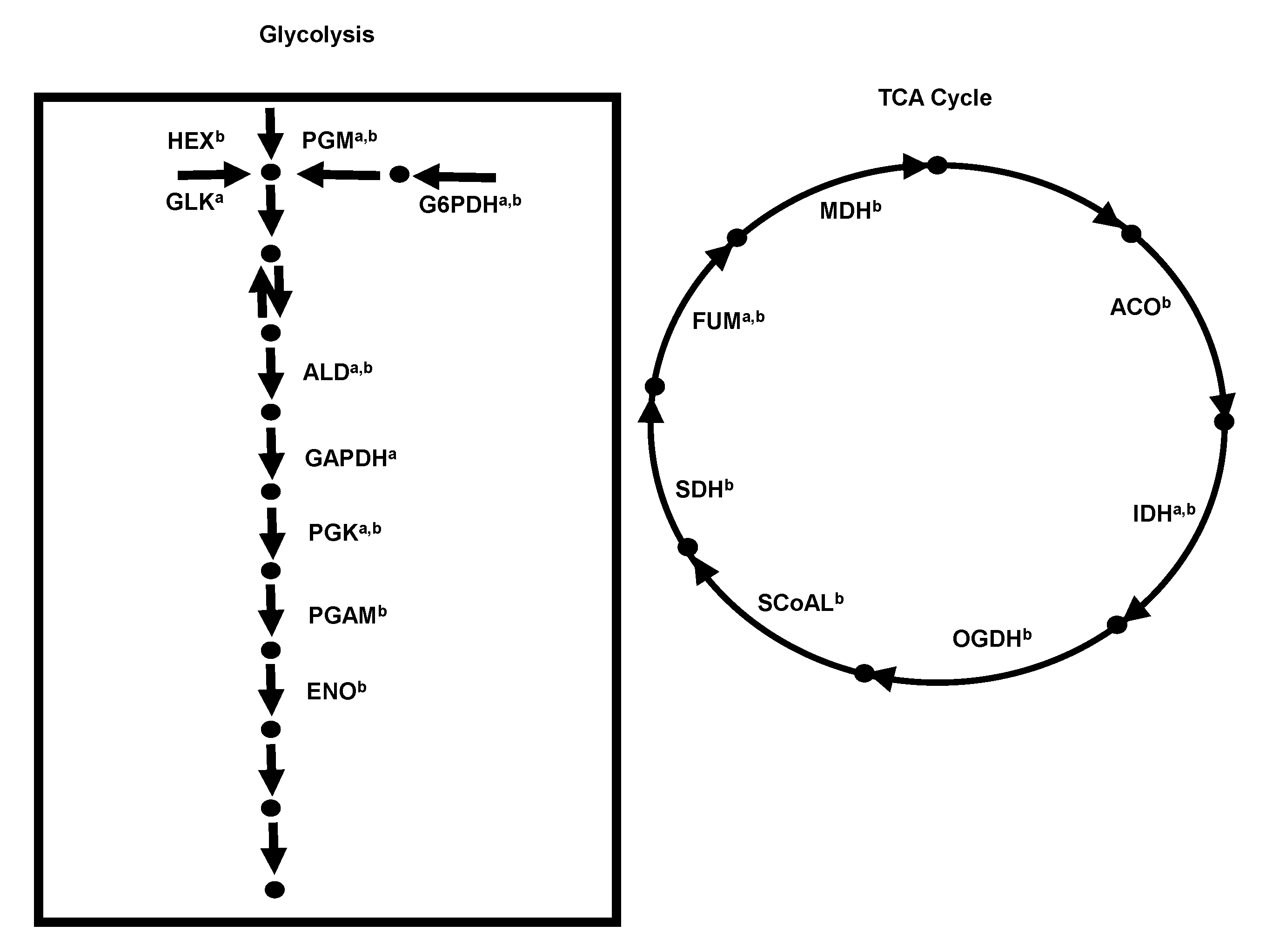

Supplement: Figure S2 — Selected CCM enzymes and their pathway location. PGM: phophoglucomutase, HEX: hexokinase, GLK: glucokinase, G6PDH: glucose-6-phosphate dehydrogenase, ALD: fructose-biphosphate aldolase, GAPDH: NADP-dependent glyceraldehyde-3-phosphate dehydrogenase PGK: phosphoglycerate kinase, PGAM: phosphoglycerate mutase, ENO: enolase, ACO: aconitase, IDH: NAD-dependent isocitrate dehydrogenase, OGDH: oxoglutarate dehydrogenase, SCoAL: succinyl-CoA ligase, SDH: succinate dehydrogenase, FUM: fumarase, MDH: NADP-dependent malate dehydrogenase. a: Enyzme activities were measured. b: Corresponding loci were sequenced. (0.13 MB TIF) [file pone.0009991.s003.tif]
