# Supplementary material for: Genetic Analysis of Central Carbon Metabolism Unveils an Amino Acid Substitution That Alters Maize NAD-Dependent Isocitrate Dehydrogenase Activity
Source: PLoS One. 2010 Apr 1;5(4):e9991. doi: 10.1371/journal.pone.0009991 (PMC2848677; doi:10.1371/journal.pone.0009991)
Supplement: File S1 — EMBL/GenBank accession numbers. (0.03 MB DOC) [file pone.0009991.s004.doc]

**File S1. EMBL/GenBank accession numbers:**

*aco* (PZB01103: BV712072-BV712108),

*ald* (PZB01715: GF097853, GF097861, GF097870, GF097878, GF097887, GF097896, GF097905, GF097914, GF097922, GF097931, GF097939, GF097948, GF097957, GF097966, GF097974, GF097982, GF097991, GF097999, GF098007, GF098015, GF098023, GF098032, GF098040, GF098047, GF098056, GF098065, GF098073, GF098080, GF098091, GF098097, GF098102, GF098108, GF098113, GF098119, GF098125, GF098131, GF098137, GF098143, GF098149, GF098155, GF098161, GF098167, GF098172; and PZB02249: GF097856, GF097864, GF097873, GF097881, GF097890, GF097899, GF097908, GF097917, GF097925, GF097934, GF097942, GF097951, GF097960, GF097969, GF097977, GF097985, GF097994, GF098002, GF098010, GF098018, GF098026, GF098035, GF098043, GF098050, GF098059, GF098068, GF098075, GF098083, GF098088, GF098094, GF098100, GF098105, GF098116, GF098122, GF098128, GF098134, GF098140, GF098146, GF098152, GF098158, GF098164, GF098170, GF098175),

*ald2* (PZB01718: GF097854, GF097862, GF097871, GF097879, GF097888, GF097897, GF097906, GF097915, GF097923, GF097932, GF097940, GF097949, GF097958, GF097967, GF097975, GF097983, GF097992, GF098000, GF098008, GF098016, GF098024, GF098033, GF098041, GF098048, GF098057, GF098066, GF098081, GF098086, GF098092, GF098098, GF098103, GF098109, GF098114, GF098120, GF098126, GF098132, GF098138, GF098144, GF098150, GF098156, GF098162, GF098168, GF098173; PZB01712: GF102221, GF102380, GF102227, GF102233, GF102238, GF102244, GF102384, GF102249, GF102255, GF102259, GF102389, GF102263, GF102268, GF102394, GF102273, GF102398, GF102403, GF102279, GF102284, GF102290, GF102296, GF102302, GF102307, GF102313, GF102319, GF102408, GF102341, GF102352, GF102357, GF102413, GF102347, GF102367, GF102361, GF102418, GF102372, GF102335, GF102376, GF102422, GF102426, GF102437, GF102431, GF102324, GF102329, GF102434; PZB02263: GF102223, GF102229, GF102240, GF102246, GF102386, GF102251, GF102257, GF102391, GF102265, GF102270, GF102275, GF102400, GF102405, GF102281, GF102286, GF102292, GF102298, GF102309, GF102315, GF102321, GF102410, GF102343, GF102354, GF102359, GF102415, GF102349, GF102369, GF102363, GF102337, GF102378, GF102424, GF102428, GF102439, GF102433, GF102326, GF102331),

*eno* (PZB01042: BV711563-BV711599),

*eno2* (PZB01067: BV711754-BV711785),

*fum* (PZB01062: BV711716-BV711753),

*g6pdh* (PZB01206: GF102379, GF102226, GF102232, GF102237, GF102243, GF102383, GF102248, GF102254, GF102388, GF102393, GF102397, GF102402, GF102278, GF102289, GF102295, GF102301, GF102306, GF102312, GF102318, GF102407, GF102340, GF102412, GF102346, GF102366, GF102417, GF102334, GF102421, GF102425, GF102430, GF102323),

*hex* (PZB01201: GF102220, GF102225, GF102231, GF102236, GF102242, GF102247, GF102253, GF102258, GF102262, GF102267, GF102272, GF102277, GF102283, GF102288, GF102294, GF102300, GF102305, GF102311, GF102317, GF102339, GF102351, GF102356, GF102345, GF102365, GF102360, GF102371, GF102333, GF102375, GF102322, GF102328)

*idh* (PZB01080: BV711814 - BV711849; PZB01689: GF097852, GF097860, GF097869, GF097877, GF097886, GF097895, GF097904, GF097913, GF097921, GF097930, GF097938, GF097947, GF097956, GF097965, GF097973, GF097981, GF097990, GF097998, GF098006, GF098014, GF098022, GF098031, GF098039, GF098046, GF098055, GF098064, GF098072, GF098079, GF098085, GF098090, GF098096, GF098101, GF098107, GF098112, GF098118, GF098124, GF098130, GF098136, GF098142, GF098148, GF098154, GF098160, GF098166; PZB02259: GF097857, GF097865, GF097874, GF097882, GF097891, GF097900, GF097909, GF097918, GF097926, GF097935, GF097943, GF097952, GF097961, GF097970, GF097978, GF097986, GF097995, GF098003, GF098011, GF098019, GF098027, GF098036, GF098044, GF098051, GF098060, GF098069, GF098076, GF098084, GF098089, GF098095, GF098106, GF098111, GF098117, GF098123, GF098129, GF098135, GF098141, GF098147, GF098153, GF098159, GF098165, GF098171, GF098176; PZB03522: GF097858, GF097866, GF097875, GF097883, GF097892, GF097901, GF097910, GF097919, GF097927, GF097936, GF097944, GF097953, GF097962, GF097971, GF097979, GF097987, GF097996, GF098004, GF098012, GF098020, GF098028, GF098037, GF098052, GF098061, GF098070, GF098077, GF098177; PZB03523: GF097867, GF097884, GF097893, GF097902, GF097911, GF097928, GF097945, GF097954, GF097963, GF097972, GF097988, GF098029, GF098053, GF098062, GF098178; and PZB03524: GF097859, GF097868, GF097876, GF097885, GF097894, GF097903, GF097912, GF097920, GF097929, GF097937, GF097946, GF097955, GF097964, GF097980, GF097989, GF097997, GF098005, GF098013, GF098021, GF098030, GF098038, GF098045, GF098054, GF098063, GF098071, GF098078),

*mdh* (PZB01077: BV711786-BV711813),

*ogdh* (PZB01092: BV711961-BV711998),

*pgam* (PZB01057: BV711682 - BV711715),

*pgk* (PZB02271: GF102224, GF102382, GF102230, GF102235, GF102241, GF102387, GF102252, GF102261, GF102392, GF102266, GF102271, GF102396, GF102276, GF102401, GF102406, GF102282, GF102287, GF102293, GF102299, GF102304, GF102310, GF102316, GF102411, GF102344, GF102355, GF102416, GF102350, GF102370, GF102364, GF102420, GF102374, GF102338, GF102429, GF102440, GF102327, GF102332, GF102436),

*pgm* (PZB01728: BV715468 - BV715501 and PZB01725: GF097855, GF097863, GF097872, GF097880, GF097889, GF097898, GF097907, GF097916, GF097924, GF097933, GF097941, GF097950, GF097959, GF097968, GF097976, GF097984, GF097993, GF098001, GF098009, GF098017, GF098025, GF098034, GF098042, GF098049,GF098058, GF098067, GF098074, GF098082, GF098087, GF098093, GF098099, GF098104, GF098110, GF098115, GF098121, F098127, GF098133, GF098139, GF098145, GF098151, GF098157, GF098163, GF098169, GF098174),

*pgm2* (PZB02247: GF102222, GF102381, GF102228, GF102234, GF102239, GF102245, GF102385, GF102250, GF102256, GF102260, GF102390, GF102264, GF102269, GF102395, GF102274, GF102399, GF102404, GF102280, GF102285, GF102291, GF102297, GF102303, GF102308, GF102314, GF102320, GF102409, GF102342, GF102353, GF102358, GF102414, GF102348, GF102368, GF102362, GF102419, GF102373, GF102336, GF102377, GF102423, GF102427, GF102438, GF102432, GF102325, GF102330, GF102435),

*scoal* (PZB01086: BV711886 - BV711922), and

*sdh* (PZB01083: BV711850-BV711885).
